# Rule breakers achieve successful shoulder balance: unraveling the myth of upper instrumented vertebrae selection criteria

**DOI:** 10.1007/s43390-024-00961-5

**Published:** 2024-09-20

**Authors:** Luke C. Drake, Peter W. D’Amore, Bailli Fontenot, Tyler A. Tetreault, Manaf Younis, Claudia Leonardi, Jaquelyn Valenzuela-Moss, Lindsay M. Andras, Michael J. Heffernan

**Affiliations:** 1 University of Mississippi Medical Center, Jackson, MS USA; 2 Children’s Hospital New Orleans, LSU Health Science Center, New Orleans, LA USA; 3 Jackie and Gene Autry Orthopaedic Center, Children’s Hospital Los Angeles, Keck School of Medicine of USC, 4650 Sunset Blvd, Mailstop #69, Los Angeles, CA 90027 USA; 4 School of Public Health, LSU Health Science Center, New Orleans, LA USA

**Keywords:** Upper instrumented vertebrae, Shoulder balance, Posterior spinal fusion, Adolescent idiopathic scoliosis

## Abstract

**Purpose:**

This study compared shoulder balance outcomes in “rule breakers” (RB) vs. “rule followers” (RF) based on commonly utilized upper instrumented vertebrae (UIV) selection guidelines.

**Methods:**

Adolescent idiopathic scoliosis (AIS) patients (Lenke 1–4) who underwent posterior spine fusion (PSF) with minimum 2-year follow-up had radiographic measurement of shoulder balance including first rib angle (FRA), T1 tilt, coracoid process height difference (CPHD), and clavicle angle (CA) at preop, postop, 6-month, 1-year, and 2-year timepoints. Postoperative outcomes were compared between RB and RF groups defined based on the UIV selection guidelines of Rose and Lenke.

**Results:**

Among 88 patients (43 RF, 45 RB), age, gender, preoperative T1 tilt, FRA, CA, and CPHD were not significantly different between groups (p > 0.05). Immediately post-surgery, the RF group had more balanced shoulders (CPHD: 11.6 mm vs. 15.7 mm, p = 0.033; CA: 2.8° vs. 3.6°, p = 0.045; FRA: 3.4° vs. 5.1°, p = 0.009; T1 tilt: 4.7° vs. 6.1°, p = 0.045). At 2 years, no difference was observed between RF vs. RB in CA (2.3 vs. 2.2°, p = 0.857) and CPHD (8.5 vs. 8.1 mm, p = 0.791). FRA and T1 tilt were higher in RB vs. RF (FRA: 4.6 vs. 2.9°, p = 0.002; T1 tilt: 5.6 vs. 3.9, p = 0.008). Shoulder balance (CPHD < 1 cm) was achieved in 73.1% of RB and 69.9% of RF at 2-year follow-up (p = 0.216).

**Conclusion:**

Adherence to commonly accepted UIV selection guidelines did not predict better shoulder balance. The RB group had worse shoulder balance immediately post-surgery, but also improved more over time. These results suggest the need to refine current UIV selection and management.

**Level of evidence:**

III.

## Introduction

Determination of level selection for the treatment of adolescent idiopathic scoliosis (AIS) has been a long-debated topic with elusive definitive answers. The end goal remains the same: a balanced spine with level shoulders and restoration of an appropriate sagittal profile. Achieving a balanced shoulder appearance is a top priority for many AIS patients as it directly affects their perception of body symmetry and overall satisfaction with the surgical outcome. To date, a standardized pathway to achieve consistent shoulder balance has not been established. Lenke et al. [1] provided a classification system for AIS and offered insight for the selection of fusion levels in AIS. Selection guidelines for the upper instrumented vertebrae (UIV) were based on relative preoperative shoulder height. Specific guidelines were as follows: proximal fusion level should be at T4 or T5 with preoperative right shoulder elevation, T4 or T3 with level shoulders, and T2 when the left shoulder is elevated preoperatively. These “rules” have been the basis for UIV selection since publication, however, the resulting shoulder balance has not been consistent, with rates of postoperative shoulder imbalance ranging from 6.4 to 56% [2–13]. The purpose of this study was to compare shoulder balance in patients treated within the traditional guidelines to those patients treated outside of the traditional rules, or “rule breakers”. We hypothesized that violation of the traditional upper instrumented vertebrae (UIV) selection rules would not significantly impact shoulder balance after posterior spinal fusion (PSF) for AIS.

## Materials and methods

We conducted an IRB-approved retrospective review of consecutive AIS patients aged 11–18 years who underwent posterior spinal fusion at a single tertiary pediatric hospital during a 24-month period. Patients with Lenke type 1, 2, 3 or 4 curve patterns who underwent selective thoracic or thoracic and lumbar spinal fusions with a minimum of 2 years post-operative clinical and radiographic follow-up were included in the study. Lenke type 5 and 6 curves as well as left-sided main thoracic curves were excluded.

**Fig. 1:**
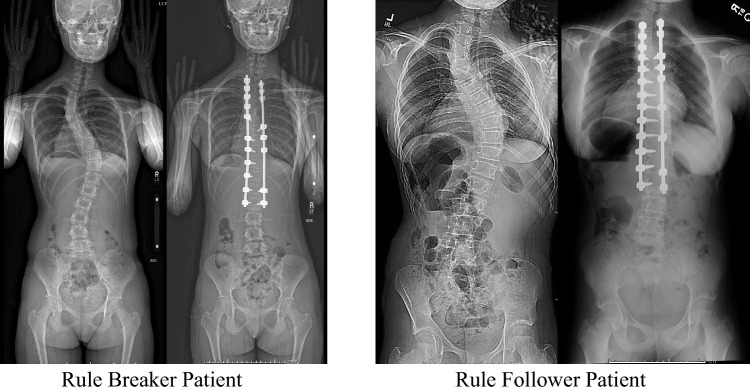
Preoperative and postoperative PA radiographs of Rule Breaker and Rule Follower showing that following current guidelines does not ensure improved shoulder balance

The classic teaching of Rose and Lenke [1] for selection of the UIV based on preoperative shoulder balance defined the study comparison groups. Patients were classified as “Rule Followers” (RF) if the UIV was selected following the rules previously described or was cephalad to the level recommended by the guidelines. Conversely, patients were classified as “Rule Breakers” (RB) if the UIV selected was caudal to the recommended guidelines (Fig. 1). Medical records were reviewed to collect demographic and clinical characteristics including age at surgery, sex, pre- and post-operative height, and spinal radiographic measurements.

### Radiographic evaluation

All patients had preoperative, immediate postoperative, 3-month, 6-month, 1-year, and 2-year follow-up with standing anteroposterior and lateral radiographs. Radiology technicians at our facility positioned the patients in an upright posture with the hands unsupported in front of the patient with slight elbow and shoulder flexion to approximately 30 degrees. All curves were classified according to the Lenke Classification [1]. Proximal thoracic (PT), main thoracic (MT), and thoracolumbar/lumbar (TL/L) curve magnitudes were recorded at the most immediate preoperative radiograph and the first erect radiograph at the postoperative visit. All radiographic measurements were performed on Synapse (Fujifilm Global Inc., Tokyo, Japan).

Four methods of shoulder balance were included in the radiographic evaluation. Coracoid process height difference (CPHD) [3, 14, 18] was defined as the measured difference between lines through the upper margin of each coracoid in relation to the horizontal plane of the radiograph. The clavicular angle (CA) [3, 15–18] was defined as the angle between a line connecting the highest point of each clavicle in relation to the horizontal plane of the radiograph. T1 tilt [3, 4, 7, 15–18] was defined as the angulation of the upper endplate of T1 to the horizontal plane of the radiograph. Positive tilt was defined as the trajectory of the line from a more superior left upper vertebral body to the more inferior right upper vertebral body of the T1 vertebra. Lastly, the first rib angle (FRA) [4, 14] was defined as the angle between the tangential line connecting the superior most aspect of the first ribs and the line horizontal to the plane of the radiograph [3, 4, 7, 15–18]. Shoulder balance was defined as a CPHD < 1 cm.

### Statistical analysis

Data were analyzed using the SAS/STAT software version 9.4 of the SAS System for PC, 2014 SAS Institute Inc. (Cary, NC). Demographic and baseline characteristics were compared between the two groups (RF vs. RB) using *χ*^2^ test for categorical variables and Student *t*-test for continuous normally distributed variables. Post intervention outcomes were compared using linear-mixed effect models (MIXED) for continuous normally distributed outcomes and generalized linear mixed models (GLIMMIX) with logit link for binary outcomes with pre-operative values included into the models as covariate and surgeon as a random effect. Measurements taken over time were analyzed as repeated measures using a compound symmetry covariance for GLIMMIX and an unstructured covariance for MIXED. Residuals were independently and normally distributed with homogenous variance where applicable. Least square mean-estimates and their standard error of the mean are presented. A 2-sided *p* < 0.05 indicated statistical significance.

## Results

Eighty-eight patients were included in the analysis including 43 RF and 45 RB. Table 1 presents the demographic, radiographic, and surgical data for the RF and RB groups. There were no significant differences in baseline demographic data between groups. Additionally, there were no differences in preoperative PT (*p* = 0.060), MT (*p* = 0.725), or TL/L (*p* = 0.065) curve magnitudes. T1 tilt (*p* = 0.726), FRA (*p* = 0.918), CPHD (*p* = 0.195), and CA (*p* = 0.382) were similar between groups. As expected, UIV distribution was more caudal in the RB group (*p* < 0.0001).

**Table 1:** Demographics and clinical characteristics of the patients

Characteristics	RF (*N* = 43)	RB (*N* = 45)	*p*-value
Age (months), mean (SD)	13.8 (1.9)	13.7 (2.3)	0.821
Height (cm), mean (SD)	161.6 (9.3)	159.4 (8.3)	0.238
PT Cobb (°), mean (SD)	24.3 (10.9)	28.7 (10.5)	0.060
MT Cobb (°), mean (SD)	54.9 (11.4)	55.8 (11.8)	0.725
TL/L Cobb (°), mean (SD)	39.8 (10.7)	34.7 (14.4)	0.065
T1 tilt^a^ (°), mean (SD)	5.9 (4.7)	6.2 (5.0)	0.726
First rib angle (°), mean (SD)	3.9 (2.4)	3.8 (3.1)	0.918
Clavicle angle^a^ (°), mean (SD)	3.0 (2.0)	2.6 (1.6)	0.382
CPHD (mm), mean (SD)	12.3 (7.5)	10.1 (7.7)	0.195
*Sex, % (n)*			0.896
Female	76.7 (33)	75.6 (34)	
Male	23.3 (10)	24.4 (11)	
*Lenke classification, % (n)*			0.620
1	81.8 (27)	76.4 (42)	
2	9.1 (3)	14.6 (8)	
3	9.1 (3)	5.4 (3)	
4	0 (0)	3.6 (2)	
*UIV, % (n)*			< 0.0001
T1	2.3 (1)	0 (0)	
T2	7.0 (3)	0 (0)	
T3	20.9 (9)	20.0 (9)	
T4	69.8 (49)	42.2 (19)	
T5	0 (0)	33.4 (15)	
T6	0 (0)	2.2 (1)	
T7	0 (0)	2.2 (1)	
*Rod type*^*1*^*, % (n)*			0.831
Cobalt chrome	38.1 (16)	34.1 (14)	
Hybrid	47.6 (20)	48.8 (20)	
Stainless steel	2.4 (1)	0 (0)	
Titanium	11.9 (5)	17.1 (7)	
*Hook used, % (n)*	48.9 (22)	60.5 (26)	0.276

*CPHD* coracoid process height difference, *MT* main thoracic, *PT* proximal thoracic, *RF* rule followers defined as fusion constructs adhering to current UIV selection guidelines or above (Rose and Lenke 2007), *RB* rule breakers, defined as fusion constructs ending below current UIV selection guidelines (Rose and Lenke 2007), *SD* standard deviation, *TL*/*L* thoracolumbar/lumbar, *UIV* upper instrumented vertebra

^a^T1 tilt and clavicle angle was not reported on one patient and rod type was not reported on 5 patients

Table 2 compares the radiographic shoulder balance indicators between the two groups at the immediate postoperative and 2 years postoperative. Significant differences were found between the groups in CPHD (*p* = 0.033), T1 tilt (*p* = 0.045), FRA (*p* = 0.009) and CA (*p* = 0.045) on the immediate postoperative films. All four measurements were smaller in the RF group compared to RB group. The 2-year postoperative measurements showed no significant difference in CPHD (*p* = 0.791) or CA (*p* = 0.857). A significant difference in T1 tilt (*p* = 0.008) and FRA (*p* = 0.002) remained at the 2-year follow-up.

**Table 2:** Outcomes^a^ measured post-surgery and at 2 years post-surgery reported as least square means (SEM) *n*

	*n*		RF (*N* = 43)		RB (*N* = 45)		*p-value*
*Post-surgery*							
T1 tilt (°)	86		4.7 (0.5)		6.1 (0.5)		0.045
First rib angle (°)	88		3.4 (0.5)		5.1 (0.4)		0.009
Clavicle angle (°)	86		2.8 (0.3)		3.6 (0.3)		0.045
CPHD (mm)	86		11.6 (2.1)		15.7 (2.0)		0.033
*2 years post-surgery*							
T1 tilt (°)	87		3.9 (0.5)		5.6 (0.5)		0.008
First rib angle (°)	85		2.9 (0.4)		4.6 (0.4)		0.002
Clavicle angle (°)	84		2.3 (0.2)		2.2 (0.2)		0.857
CPHD (mm)	85		8.5 (1.2)		8.1 (1.2)		0.791

*CPHD* coracoid process height difference, *LSM* least square mean, *RF* rule followers defined as fusion constructs adhering to current UIV selection guidelines or above (Rose and Lenke 2007), *RB* rule breakers, defined as fusion constructs ending below current UIV selection guidelines (Rose and Lenke 2007), *SEM* standard error of the mean

^a^Model included pre surgery values as covariates and surgeon as a random effect

**Fig. 2:**
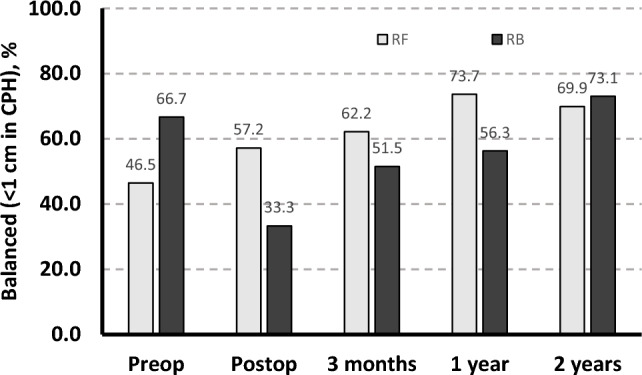
Percentage of patients with balanced shoulders, defined as < 1 cm in coracoid process height (CPH) difference between the two shoulders, in rule follower (RF, light gray) and rule breakers (RB, black) patients

The initial postoperative radiographic shoulder balance for RF was 57.2% and for RB was 33.3% that improved to 69.9% and 73.1%, respectively at the 2-year follow-up (Fig. 2). The RF group showed improvement in shoulder balance with surgery, from 46.5% preoperatively to 57.2% at the immediate postoperative time point. Conversely, shoulder balance worsened with surgery in the RB group, from 66.7% preoperatively to 33.3% at the initial postoperative time point. However, significant improvement was seen until final follow-up, where shoulder balance was achieved in 73.1%. Overall, the effect of time was found to play a significant role in shoulder balance improvement for both groups (*p* = 0.014) and no difference was ultimately found in shoulder balance between groups (*p* = 0.216).

## Discussion

Following the development of a new classification system in 2007 by Lenke et al. [1], surgeons have routinely utilized the guidelines for UIV selection in AIS cases. However, shoulder balance remains unreliable with rates of imbalance as high as 56% published in the literature [12]. In our study, shoulder balance was achieved in ≥ 70% by 2-year follow-up. Interestingly, only 33% of the RB group had shoulder balance at the initial postoperative timepoint. In fact, all radiographic measures of shoulder balance were superior in the RF group compared to the RB group in the early postoperative period. However, the RB group showed significant improvement in shoulder balance parameters over time and at 2-year follow-up there was no difference in shoulder balance between the groups. Our results suggest that non-adherence to commonly utilized UIV guidelines does not preclude achievement of acceptable shoulder balance at 2-year follow-up.

Although UIV selection is a point of focus for surgeons and shoulder balance is considered an important outcome following surgery for AIS, the relationship between UIV selection and shoulder balance remains poorly understood. Surgeons have utilized the Lenke guidelines to achieve predictable outcomes**.** However, in contrast to commonly held dogma, Brooks et al. [13] found that patients with a UIV at T2 or T3 experienced more postoperative shoulder imbalance when compared to patients with T4 chosen as the UIV. Furthermore, Tang et al. [9] evaluated the effect of cosmetic shoulder balance in relation to shorter segment fusions for Lenke 1 curves. Although they did not consider radiographic measures, 93% of their patients had clinically acceptable shoulder balance at 2 years regardless of a long or short fusion strategy. These findings mirror our results, questioning the role of UIV selection in achieving postoperative shoulder balance.

Others have attempted to develop alternative methods of UIV selection. Ilharreborde et al. [7] published selection criteria based on the amount of rigidity of the PT curve on anterior–posterior (AP) radiographs, AP bending radiographs, T1 tilt, and pre-operative shoulder balance. Torbish et al. [19] attempted to simplify UIV selection based on the Lenke classification and pre-operative shoulder balance. Bjerke et al. [10] compared the three strategies (Lenke, Ilharreborde and Torbish) for UIV level selection and found no significant differences in shoulder balance, regardless of the strategy utilized. In our cohort, although the RB group had worse initial shoulder balance, we found that breaking the rules of UIV selection did not portend worse shoulder balance at 2-year follow-up. Looking at our results in conjunction with existing literature, it is reasonable to conclude that the goal of achieving shoulder balance should not be the driving force in UIV decision making.

The impact of time on shoulder balance was another notable finding of our study. The RF group had worse shoulder balance preoperatively which linearly improved postoperatively. In contrast, the RB group’s shoulder balance initially worsened in the immediate postoperative period but showed significant improvement over time. Ultimately, both groups achieved similar shoulder balance at 2-year follow-up. In a cohort of 30 consecutive AIS patients with Lenke 2 curves, Elfiky et al. [16] showed that some patients with unfused proximal thoracic curves had mild improvement in their shoulder balance parameters postoperatively. While the exact reasons for this gradual improvement in shoulder balance remain somewhat mysterious, several potential mechanisms might be at play. One possibility is that the surrounding soft tissues slowly adjust to the new spinal alignment after surgery, allowing the shoulders to settle into a more balanced position over time. Additionally, the unfused segments above the upper instrumented vertebrae might naturally shift their alignment in response to the corrected portion, similar to the behavior seen in lumbar curves following selective thoracic fusion. Based on our results, surgeons can expect radiographic shoulder balance to gradually improve with time regardless of the UIV selection strategy.

Despite the important findings of this study, there are several limitations that need to be acknowledged. This was a single center, retrospective study with limitations intrinsic to retrospective investigations. Most of the patients in the study had Lenke 1 curves with a smaller cohort of Lenke 2, 3, and 4 type curves which limits the generalizability of this study for those underrepresented curve types. Further, Lenke type 5 and 6 curves were excluded due to the major deformity being within the lumbar spine and thus exerting less of direct influence over shoulder balance and left sided main thoracic curves were excluded as an atypical curve pattern**.** Our study focused on radiographic shoulder balance and did not include assessment of clinical appearance. Additionally, the role of chronologic age was not assessed in relation to shoulder balance improvement over time. Further study looking at the impact of patient age at time of surgery would be beneficial to understand improved shoulder balance with time. Finally, patient reported outcomes were not available for these patients.

In conclusion, our findings suggest that breaking the recommended Lenke guidelines for UIV selection has the potential to achieve similar radiographic shoulder balance when compared to UIV selections that follow existing guidelines. The RB group had remarkably worse shoulder balance in the immediate postoperative period when compared to the RF group. However, the RB group also experienced a more dramatic improvement in shoulder balance with time. This information can be utilized to counsel families and manage both surgeon and patient expectations in the perioperative period. To date, there are no UIV selection criteria that lead to superior postoperative shoulder balance. Future work should focus on further evaluation of UIV selection criteria with the goal of establishing a clear pathway to consistent postoperative shoulder balance.

## Data Availability

The dataset utilized for this study is available upon request.
